# Early intraoperative hypotension and its associated factors among surgical patients undergoing surgery under general anesthesia: An observational study

**DOI:** 10.1016/j.amsu.2021.102835

**Published:** 2021-09-11

**Authors:** Netsanet Temesgen, Efrem Fenta, Chernet Eshetie, Moges Gelaw

**Affiliations:** Debre Tabor University, Ethiopia

**Keywords:** Early intraoperative hypotension, General anesthesia, Surgical procedures

## Abstract

**Background:**

Early intraoperative hypotension (eIOH) is a common complication of general anesthesia and is significantly associated with postoperative morbidity and mortality. The incidence of eIOH was high, especially in resource-limited settings. Identifying the factors associated with the occurrence of eIOH might allow avoidance and planning of a timely treatment of it.

**Objective:**

To assess the incidence of early intraoperative hypotension and its associated factors among surgical patients undergoing Surgical procedures under general anesthesia at **XX** Comprehensive Specialized Hospital, North-central Ethiopia, 2021.

**Methods:**

A total of 424 surgical patients under general anesthesia were included in this prospective observational study. The data were collected by a structured questionnaire. Variables with p-values of less than 0.2 in the bivariable logistic regression were fitted to multivariable logistic regression. Data was presented in odds ratios with a 95% confidence interval. Descriptive statistics were used to summarize data.

**Results:**

The incidence of early intra-operative hypotension (eIOH) was 21.2%. In this study older age (age≥ 60 years) (AOR: 2.063 (95% CI;1.194, 3.563)), ASA physical status (AOR: (II2.259 (95% CI;1.229, 4.153)), III(AOR: 2.810 (95% CI;1.319, 5.986)), a BMI of 25–29.9 kg/m2 (AOR: 2.098 (1.128, 3.901), a BMI of ≥30 kg/m2 (AOR: 3.090 (95% CI;1.324, 7.210)), emergency surgical procedures (AOR: 2.215 (95% CI;1.287, 3.810)), the estimated blood loss greater than 500 ml (AOR: 2.510 (95% CI;1.478, 4.261)) were found to be independent factors of eIOH.

**Conclusion:**

This study revealed that the incidence of eIOH was high (21.2%). Older age, ASA II and III, BMI ≥25, emergency surgical procedures, and a significant amount of blood loss (EBL ≥500 ml) were the main predictors of an increased occurrence of eIOH.

## Highlights

• Early intraoperative hypotension is associated with postoperative morbidity and mortality.
• Emergency surgical procedures, and a significant amount of blood loss are the main predictors of an increased occurrence of eIOH.
• Thyroidectomy has a relatively short duration and less amount of estimated blood loss when compared to major surgical procedures.
• The occurrence of eIOH after general anesthesia for general surgery is a common problem.
• Arterial hypotension in patients undergoing surgery under general anesthesia usually described by the very general term intraoperative hypotension.

## 1 Introduction

There were a variety of definitions for eIOH. Hypotension was defined as either mean arterial blood pressure (MAP) decrease of >40% and MAP <70 mm Hg or MAP<60 mm Hg. A decline in arterial blood pressure of 30% from the preoperative reading value is another definition of it. The other definition of eIOH is a decrement in systolic blood pressure SBP less than 90 mmHg.

In this study, early intraoperative hypotension (eIOH) was defined as a decrease in systolic blood pressure SBP< 90 mmHg, which was supported by recent literature that might lead to similar conclusions for having postoperative IOH-related risk of morbidity and mortality in contrast to a decline of arterial blood pressure from a baseline [[1], [2], [3]].Early intraoperative hypotension has been associated with adverse outcomes like kidney failure, myocardial injury, and mortality. Therefore prevention of early IOH may be a key in improving patient outcomes [4].

Arterial hypotension in patients undergoing surgery under general anesthesia usually described by the very general term ‘intraoperative hypotension (IOH) is highly prevalent and associated with unfavorable patient outcomes. An arterial blood pressure decline below the lower limit of the vascular autoregulation curve might lead to ischemia of vital organs (i.e. Heart, Brain, and Kidney) [5]. An association between intraoperative hypotension and adverse outcomes remains debatable and there is no consensus on the lowest acceptable intraoperative blood pressure. Data indicate a higher mortality rate in the elderly when there is a decrease in SBP of 40–45% from the preoperative baseline. However, recent reports indicated that intraoperative hypotension contributes to troponin elevation and is associated with increased 30-day mortality [6].

In recent years, evidences are growing that the development of IOH is associated with adverse postoperative outcomes in terms of both morbidity and mortality. In a large observational study in 10,440 adult patients undergoing non-cardiac surgery increased durations of IOH (defined by MAP thresholds between 50 and 80 mmHg) were strongly associated with 30-day mortality. In another large retrospective cohort study in 18,756 patients undergoing non-cardiac surgery these findings again confirmed the case, as an increase in 30-day mortality was found for patients with either SBP <70 mmHg, MAP <49 mmHg, or DBP <30 mmHg for at least 5 min.

Multiple other studies have shown the association between IOH and postoperative morbidity in terms of myocardial and acute kidney injury and possibly for the occurrence of ischemic stroke too. Importantly, these studies also demonstrated that even short instances of IOH (in some studies as short as 1 min) were associated with adverse outcomes. For example in one of these studies performed in 33,330 patients underwent non-cardiac surgery, the odds ratio of postoperative myocardial injury and kidney injury when MAP was lower than 55 mmHg for only 1–5 min. Moreover, even short periods of post-induction hypotension before surgical incision are associated with postoperative acute kidney injury [4].

Intraoperative blood pressures, MAP less than 55 mmHg was associated with the development of acute kidney injury, myocardial injury, and cardiac complications [7].

Arterial hypotension during the early phase of anesthesia can lead to adverse outcomes such as a prolonged postoperative stay or even death. Predicted hypotension during anesthesia induction is complicated by its diverse causes [8].

The occurrence of eIOH after general anesthesia for general surgery is a common problem, especially in resource-limited settings like ours. Knowing the incidence and associated factors causing eIOH helps urgent need of safety and treatment in the service of anesthesia and surgery. This research can also help as formulate baseline information for future researches in Ethiopia. This study will help to determine the incidence and factors associated with eIOH in surgical procedures under general anesthesia in **XX** Comprehensive Specialized Hospital.

## 2 Methods and materials

### 2.1 Study area and period

XX compressive specialized Hospital is a public Hospital established in 1934 and located in South Gonder Zone, Amara region 667 km northwest of Addis Ababa, the capital city of Ethiopia at Debre Tabor town which is 97 km to the southwest of Bahir Dar, the capital city of Amara region. According to the 2007 census, the total population of this town was 155,596. The study was conducted in Debre Tabor Town at XX Referral Hospital 2021 GC. It has one referral hospital, and its altitude of 2706 m or 8878 feet above sea level [9]. The hospital has 6 operation rooms which are divided into specialties i.e. 2 rooms for general surgery, 1 room for obstetrics, 1 room for gynecological procedures, and 2 rooms for orthopedics. So, the study participants were taken from any surgery performed with induction agents as propofol, ketamine, and thiopental with intubating relaxation by suxamethonium, and the study would have been carried out from January 1 to April 25/2021 GC.

### 2.2 Study design

A prospective Observational study design was used, this study has been registered in a research registry http://www.researchregistry.com with a registry number **6911**.

Our work is fully compliant with the STROCSS criteria www.strocssguideline.com, in which a completed STROCSS checklist stating the page numbers(Agha R, Abdall-Razak A, Crossley E, Dowlut N, Iosifidis C and Mathew G, for the STROCSS Group. The STROCSS 2019 Guideline: Strengthening the Reporting of Cohort Studies in Surgery. International Journal of Surgery 2019; 72:156–165).

### 2.3 Population

#### 2.3.1 Source of population

All elective and emergency surgical patients who would undergo surgery under general anesthesia.

#### 2.3.2 Study population

All elective and emergency surgical patients who would undergo surgery under general anesthesia met the inclusion criteria during the study period.

### 2.4 Variables

#### 2.4.1 Dependent variable

Early intraoperative Hypotension (Yes/No)

#### 2.4.2 Independent variables

Section 1**: demographic variables;** Age, sex, ASA physical status, BMI (Kg/m^2^), patient has any co morbidity, Use of chronic medications
Section 2**: Surgery-related variables:** Surgeon experience, Planned procedure, Urgency of the procedure, Estimated blood loss, Intraoperative positioning, Duration of surgery,
Section 3**: Anesthetic related variables:**Anesthetist experience, Premedication, Preoperative fluid intake, Intravenous induction agent, Analgesics used, Muscle relaxant used, ETT size, Number of Intubation attempts, Intraoperative fluid, Intraoperative vasoactive agent.

### 2.5 Sampling size determination and sampling technique

#### 2.5.1 Sampling size determination

The sample size was determined by taking the following assumption; since there is no previous study conducted in Ethiopia. We will assume the proportion is 50%, confidence interval of 95% and margin of error of to be tolerated 0.05%.The sample size to be taken for the study was determined using the formula.

n=z2(p)∗(1−p)/d2

Whereas, n = sample size; Z = confidence interval (1.96) for 95% confidence interval; P = estimated prevalence (0.5); d = margin of sampling error to be tolerated (0.05).

n= (1.96)^2^0.5(1–0.5)/0.05^2^–385, by adding 10% non-response rate the total sample size was 424.

#### 2.5.2 Sampling technique

The final study subjects were chosen by a consecutive convenience sampling technique. The study units that happen to be available at the time of data collection were the participants of the study.

### 2.6 Inclusion and exclusion criteria

#### 2.6.1 Inclusion criteria

All age ≥18years patients who came to OR for elective and emergency surgery under GA during the study period were included.

#### 2.6.2 Exclusion criteria

✓ Shocked patient
✓ Pregnant mother
✓ Cardiac patient
✓ ASA IV and more
✓ Two or more intubation attempts

### 2.7 Data collection technique and instrument

Data was collected using structured questionnaires prepared in English. Data was collected by trained anesthetists. the study tool includes demographic data of the study participants (age, sex, ASA physical status, BMI (kg/m^2^), patient has any comorbidity, use of chronic medications), surgery-related data of the study participants (surgeon experience, planned procedure, urgency of the procedure, estimated blood loss, intraoperative positioning, duration of surgery), and anesthetic related data of the study participant (anesthetist experience, premedication, preoperative fluid intake, intravenous induction agent, analgesics used, muscle relaxant used, ETT size, number of intubation attempts, intraoperative fluid, intraoperative vasoactive agent).This tool was developed after searching various literatures related to this topic [2,10,5,11,12,13].

### 2.8 Ethical considerations

Ethical approval was obtained from the Ethical Review Committee of **XX**U. A formal letter was written to **XX**CSH. Permission was asked from the responsible Authorities of DTCSH. Written consent was obtained from each participant after giving brief explanation about the objective (purpose) and benefit of the study as clearly as possible to the study participants.

### 2.9 Data quality control and assurance

To ensure the quality of data, a pre-test of the questionnaire was performed on 5% of study populations who fulfill the inclusion criteria at **CC** specialized and teaching hospital. The completed questionnaire would have been submitted and reviewed daily to avoid loss of data. Close supervision and daily information exchange including by telephone would have been used as a means to correct problems during the data collection period. Data consistency and completeness would have been made throughout the data collection, data entry, and analysis.

### 2.10 Data processing and analysis

The data were coded and entered and in SPSS version 25 statistics software for analysis. The association between the dependent variable (eIOH) with independent variables was analyzed by bivariable logistic regression and multivariable logistic regression. Variables with p-values of less than 0.2 in the bivariable logistic regression were fitted to multivariable logistic regression. Data was presented in Odds ratios with a 95% confidence interval. Descriptive statistics were be used to summarize data. Tables were used to present the data.

### 2.11 Operational definitions

Early intraoperative hypotension: Is a systolic blood pressure less than 90 mmHg during the first 30min of surgery [5].

Baseline blood pressure: The measurement of arterial blood pressure taken before the induction of anesthesia.

## 3 Results

### 3.1 Demographic and patient-related data of the study participants

Four hundred twenty-four adult elective and emergency surgical patients under general anesthesia took part in this study. Among the participants, 59.9% were male. In this study, 70.75% were ASA I, and the majority (85.85%) did not have co morbidities (Table 1).

**Table 1:** Demographic and patient-related data in patients undergoing surgical procedures under general anesthesia at **XX** Comprehensive Specialized Hospital, 2021 (N = 424).

Variables		Presence of eIOH		Total, No. (%)
		Yes, No. (%)	No, No. (%)	
Age (Years) (n = 424)	19–59	48(11.32%)	261(61.55%)	309 (72.88%)
	≥60	42(9.9%)	73(17.22%)	115(27.12%)
Sex	Male	60(14.15%)	194(45.75%)	254(59.9%)
	Female	30(7%)	140(33%)	170(40.1%)
ASA Physical status	ASA I	46(10.85%)	254(59.9%)	300(70.75%)
	ASA II	27(6.37%)	54(12.7%)	81(19.11%)
	ASA III	17(4%)	26(6.13%)	43(10.14%)
BMI (Kg/m2)	18.5–24.9	49(11.56%)	270(63.7%)	319(75.23%)
	25–29.9	28(6.6%)	47(11%)	75(17.69%)
	≥30	13(3.08%)	17(4%)	30(7.08%)
Presence of co morbid disease	NO	72(17%)	292(68.87%)	364(85.85%)
	Yes	18(4.24%)	42(9.9%)	60(14.15%)
Use of chronic anti-hypertension medications	NO	75(17.7%)	299(70.5%)	374(88.2%)
	Yes	15(3.54%)	35(8.25%)	50(11.8%)

### 3.2 Surgery-related data of study participants

From 424 surgical patients majority of the surgery constituents abdominal and elective surgical procedure accounts 349(82.32%) and 307(72.4%) respectively and incidence of early intraoperative hypotension during emergency surgery accounts 41(9.67%) (Table 2).

**Table 2:** Surgery-related data in patients undergoing surgical procedures under general anesthesia at **XX** Comprehensive Specialized Hospital, 2021 (N = 424).

Variables		Presence of eIOH		Total
		Yes	no	
Type of surgery	Abdominal	69(16.27%)	280(66%)	349(82.32%)
	Gynecological	15(3.53%)	36(8.46%)	51(12.02%)
	Orthopedics	6(1.4%)	18(4.24%)	24(5.66%)
Urgency	Elective	49(11.56%)	258(60.85%)	307(72.4%)
	Emergency	41(9.67%)	76(17.9%)	117(27.6%)
Positioning	Supine	72(17%)	283(66.7%)	355(83.73%)
	Lateral	18(4.24%)	51(12%)	69(16.27%)
EBL	<500 ml	47(11.08%)	250(59.96%)	297(70.05%)
	≥500 ml	43(10.14%)	84(19.81%)	127(29.95%)
Surgeon experience (years	0–3	12(2.8%)	63(14.9%)	75(17.7%)
	4–6	60(14.1%)	183(43.2%)	243(57.3%)
	≥7	18(4.3%)	88(20.7%)	106(25%)
Duration of surgery (minute	<60min	38(8.96%)	152(3.85%)	190(44.81%)
	≥60min	52(12.27%)	182(42.92%)	234(55.19%)

### 3.3 Anesthetic-related data of the study participants

The majority of study participants (41.04%) were induced ketamine. The majority of study participants (89.15%) did not take vasopressors, and most of surgical patients **(**67.69%) took less than 1000 ml of crystalloids in the intraoperative period (Table 3).

**Table 3:** Anesthetic-related data in patients undergoing surgical procedures under general anesthesia at **XX** Comprehensive Specialized Hospital, 2021 (N = 424).

Variables		Presence of eIOH		Total
		Yes	No	
Preoperative fluid intake	<1000 ml	60(14.15%)	222(52.36%)	282(66.51%)
	1000 ml-2000	18(4.24%)	89(21%)	107(25.24%)
	2000–3000 ml	12(2.83%)	23(5.42%)	35(8.25%)
Aspiration prophylaxis	NO	36(8.5%)	109(25.7%)	145(34.2%)
	Metoclopramide	54(12.7%)	225(53%)	279(65.8%)
Intraoperative analgesics used	Opioids	51(12%)	159(37.5%)	210(49.52%)
	Diclofenac	16(3.77%)	69(16.28%)	85(20.05%)
	Opioids & Diclofenac	23(5.43%)	106(25%)	129(30.43%)
IV induction agent	Ketamine	36(8.49%)	138(32.54%)	174(41.04%)
	Propofol	30(7.07%)	73(17.2%)	103(24.3%)
	Ketofol	24(5.66%)	123(29%)	147(34.66%)
Muscle relaxant used	Suxamethonium	18(4.24%)	81(19.1%)	99(23.35%)
	Suxamethonium & Vecuronium	72(16.98%)	253(59.67%)	325(76.65%)
ETT size	6.5	18(4.24%)	74(17.45%)	92(21.7%)
	7.0	42(9.9%)	173(40.8%)	215(50.7%)
	7.5	30(7.07%)	87(20.5%)	117(27.6%)
Number of intubation attempts	Single	72(16.98%)	282(66.5%)	354(83.5%)
	Multiple	18(4.24%)	52(12.26%)	70(16.5%)
Amount intraoperative fluid intake	<1000 ml	54(12.7%)	233(54.95%)	287(67.69%)
	1000–2000 ml	36(8.5%)	101(23.8%)	137(32.31%)
Vasoactive agent used	No	78(18.4%)	300(70.75%)	378(89.15%)
	Yes	12(2.83%)	34(8.02%)	46(10.85%)
Anesthetist experience (years	0–3	12(2.83%)	52(12.26%)	64(15.09%)
	4–6	60(14.2%)	244(57.5%)	304(71.7%)
	≥7	18(4.24%)	38(8.97%)	56(13.21%)

### 3.4 The incidence of early intraoperative hypotension

**Fig. 1:**
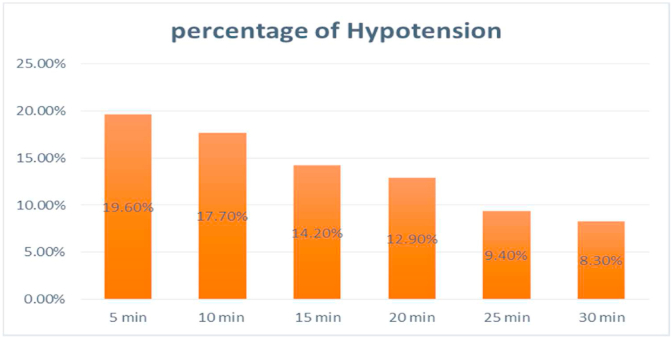
The incidence of early intraoperative hypotensionin patients undergoing surgical procedures under general anesthesia at **XX** Comprehensive Specialized Hospital, 2021 (N = 424).

In this study, Early intraoperative hypotension was found in 90 (21.2%) of study participants. The majority of (19.6%) of the eIOH occurred within 5 min after the induction of anesthesia (Fig. 1).

### 3.5 The variables associated with the eIOH

In this study older age 2 times (age≥ 60 years) (AOR: 2.063 (95% CI;1.194, 3.563)) more likely to develop eIOH, ASA II patients had 2.2 times (AOR: 2.259 (95% CI;1.229, 4.153)) more likely to develop eIOH as compared to ASA I patients, ASA III patients were 2.8 times (AOR: 2.810 (95% CI;1.319, 5.986)) more likely to develop eIOH when compared to ASA I patients, a BMI of 25–29.9 kg/m^2^ was 2 times (AOR: 2.098 (1.128, 3.901) more likely to develop eIOH when compared with patients having normal BMI, a BMI of ≥30 kg/m^2^ was 3 times (AOR: 3.090 (95% CI;1.324, 7.210)) more likely to develop eIOH when compared with patients having normal BMI, emergency surgical procedures were 2.2 times (AOR: 2.215 (95% CI;1.287, 3.810)) more likely to develop eIOH when compared with elective surgical procedures, the estimated blood loss greater than 500 ml was 2.5 times (AOR: 2.510 (95% CI;1.478, 4.261)) more likely to develop eIOH when compared with patients having an estimated blood loss less than 500 ml (Table 4).

**Table 4:** Variables associated with the early intraoperative hypotensionin patients undergoing surgical procedures under general anesthesia at **XX** Comprehensive Specialized Hospital, 2021 (N = 424).

Variables		Presence of eIOH		COR (95% CI)	AOR (95% CI)	P-value
		Yes	No			
Age (Years)	19–59	48	261	1	1	
	≥60	42	73	3.128 (1.919,5.100)	2.063 (1.194, 3.563)	0.009
ASA Physical status	ASA I	46	254	1	1	
	ASA II	27	54	2.761 (1.579,4.827)	2.259 (1.229, 4.153)	0.009
	ASA III	17	26	3.610 (1.816,7.178)	2.810 (1.319, 5.986)	0.007
BMI (Kg/m^2^)	18.5–24.9	49	270	1	1	
	25–29.9	28	47	3.283 (1.879,5.736)	2.098 (1.128, 3.901)	0.019
	≥30	13	17	4.214 (1.925,9.226)	3.090 (1.324, 7.210)	0.009
Urgency	Elective	49	258	1	1	
	Emergency	41	76	2.840 (1.745,4.624)	2.215 (1.287, 3.810)	0.004
EBL (ml)	<500	47	250	1	1	
	≥500	43	84	2.723 (1.682,4.408)	2.510 (1.478, 4.261)	0.001

## 4 Discussion

Early intraoperative hypotension is associated with postoperative morbidity and mortality(8,9,19,21–23). In this study, we found that the incidence of early intraoperative hypotension within 30 min after general anesthesia in major surgery was 21.2%. In line with our finding, a study conducted by Su et al. [5] found that the incidence of eIOH was 24.7%. Incongruent to our result, Kalezic et al. [11] reported that the incidence of IOH was lower (6.5%) as compared to our finding. This might be due to differences in the type of surgery (thyroid surgery). Thyroidectomy has a relatively short duration and less amount of estimated blood loss when compared to major surgical procedures (e.g. abdominal) in which the operation can last a longer duration (several hours) and might have a significant blood loss. Jor et al., and Cheung et al. observed that there was a higher incidence of IOH (55.4% [14], 36.5% [10]) as compared to our findings. This difference might be due to most of the patients were induced with propofol.

In this study, early intraoperative hypotension (eIOH) was defined as a decrease in systolic blood pressure SBP<90 mmHg. Some recent studies support this approach, revealed that definitions of eIOH using SBP less than 90 mmHg might lead to similar conclusions for having postoperative IOH-related risk of morbidity and mortality when compared with a lower arterial blood pressure from baseline (SBP >30% decline from preoperative arterial BP) [2,3].

In this study older age (age≥ 60 years), ASA physical status (II, III), BMI (>25 kg/m2), emergency surgical procedures, estimated blood loss greater than 500 ml were found to be independent factors of an increased occurrence of eIOH.

In this study ASA physical status (II, III) was found to be an independent factor of an increased occurrence of eIOH. In line with this study, Su et al. [5] revealed that ASA physical status affects the occurrence of eIOH. In contrast to our result, a study conducted by Stojanovic et al. [11] ASA physical status did not affect the occurrence of eIOH, this might be due to they included relatively younger patients.

In this study older age (age ≥60 years) was an independent risk factor for eIOH. Similarly, a study conducted by Su et al. [5] found that older age was associated with an increased occurrence of eIOH. Another recent study conducted by Reich et al. [15] and Cheung et al. [14] also revealed that there was an association between eIOH and ages of >65 and > 50 years respectively.

In our study emergency surgical procedures were associated with the occurrence of eIOH. In line with this study, Su et al. revealed that emergency surgical procedures were the predictors of eIOH(6). This finding might reflect the fact that patients undergoing emergency surgical procedures compared with elective surgical procedures are more likely to develop cardiovascular dysfunction.

In this study BMI >25 kg/m2 was found to be independent factors of an increased occurrence of eIOH. In contrast to our result Kalezic et al. [11] revealed that BMI did not affect the occurrence of eIOH, the possible explanation for this difference could be thyroidectomy requires a relatively short duration of time and less amount of estimated blood loss in contrast with our study that most of the surgical procedures were abdominal surgeries, which can last for a prolonged period and having a significant blood loss.

The definition of hypotension is the main problem of this study. The variability of definitions of eIOH, significantly impacts comparisons and interpretations of the results of studies concerning the occurrence of eIOH(2). The other limitation could be this study does not take into consideration the duration of hypotension, and managing eIOH according to the available medical equipment and drugs (e.g. adrenaline is a frequently administered drug for hypotension) could be the limitations of this study.

## 5 Conclusion

The incidence of eIOH was high (21.2%). Older age, ASA II and III, BMI ≥25, emergency surgical procedures, and a significant amount of blood loss (EBL ≥500 ml) were the main predictors of an increased occurrence of eIOH. Identifying those factors might allow the avoidance and planning of a timely treatment of hypotensive episodes during general anesthesia.

## Ethical approval

Ethical clearance was obtained from Debre tabor University research review committee.

## Sources of funding

No funding is required.

## Author contribution

All authors equally contributed to the study concept or design, data searching, data analysis or interpretation, writing the paper.

## Guarantor

Mr. Netsanet Temesgen.

## Consent

Not applicable for that.

## Funding

None.

## Declaration of competing interest

The authors declared that there is no conflict of interest.

## Acronyms and abbreviations

AOR: Adjusted odds ratio
ASA: American Societies Anesthesiologist
CI: Confidence interval
COR: Crud odds ratio
**XX**CSH: **XX** Comprehensive Specialized Hospitals
eIOH: early Intraoperative Hypotension
EBL: Estimated blood loss
GA: General Anesthesia
MAP: Mean Arterial Blood Pressure
OR: Operation Room
SBP: Systolic Blood Pressure
SPSS: Statistical Package for Social Sciences
